# Association between usage intensity of short video platforms and altered brain function: a resting-state functional magnetic resonance imaging study

**DOI:** 10.3389/fnhum.2026.1786568

**Published:** 2026-05-21

**Authors:** Siwei He, Shixiong Tang, Dayi Liu, Zhiyuan Chen, Qinyu Zou, Yicheng Long

**Affiliations:** 1 Chongqing Shapingba Mental Health Center, Chongqing, China; 2 Department of Radiology, The Second Xiangya Hospital, Central South University, Changsha, Hunan, China; 3 Department of Psychiatry and Psychology, Jiangmen Central Hospital, Jiangmen, Guangdong, China; 4 Department of Psychiatry, National Clinical Research Center for Mental Disorder, The Second Xiangya Hospital, Central South University, Changsha, Hunan, China; 5 School of Medical Imaging, Changsha Medical University, Changsha, Hunan, China

**Keywords:** connectomics, fMRI, functional brain network, functional connectivity, graph theory, short videos

## Abstract

**Background:**

The potential negative influences of short video platforms (SVPs) usage on mental health have been attracting increasing attention in recent years. This study aimed to investigate the possible effects of SVP usage on brain functions using the resting-state functional magnetic resonance imaging (fMRI) methods.

**Methods:**

Resting-state fMRI data were acquired from a total of 55 young healthy adults. Based on self-reported daily usage time of SVPs, these participants were divided into a lower SVP usage (SVP-) group (< 1 h per day, *n* = 20) and a higher SVP usage (SVP+) group (≥1 h per day, *n* = 35). Between-group comparisons of functional brain measures were performed across multiple spatial levels.

**Results:**

At the single-edge level, the SVP + group showed significantly increased functional connectivity (FC) across many edges linking most major brain networks, including sensorimotor, visual, auditory, subcortical, default-mode, attention, and cingulo-opercular networks. Network-level analyses confirmed this widespread hyperconnectivity, with particularly robust increases within sensorimotor, auditory, subcortical, and cingulo-opercular networks after multiple comparisons correction. Voxel-wise analyses revealed higher fractional amplitude of low-frequency fluctuations (fALFF) in the left precentral gyrus and lower fALFF in the right frontal lobe in the SVP + group. Global topological analysis indicated that the SVP + group had significantly higher global efficiency, local efficiency, and clustering coefficient, as well as lower characteristic path length, suggesting an altered network topology.

**Conclusion:**

This multi-level fMRI study suggests that a relatively higher-intensity SVP use is associated with an altered pattern of brain functional organization, characterized by widespread hyperconnectivity across most major brain networks, localized spontaneous activity alterations in sensorimotor regions, and an altered topology at the global level. These findings highlight the importance of considering potential impacts of SVP usage on brain functioning, and calls for future larger-sample and longitudinal studies to further understand such relationships.

## 1 Introduction

In recent years, the popularity and availability of short video platforms (SVPs) (e.g., TikTok) have been significantly growing worldwide (Liu et al., 2024; Gao et al., 2025). In China, for instance, the number of SVP users has reached more than 800 million by 2020, and the average daily usage time was estimated at around 2 hours (Shi et al., 2024; Guo and Chai, 2024; Wu et al., 2023). Such rapid growth has raised wide concerns about the risks of SVP overuse and potential related social issues. Many previous studies have suggested that a higher intensity of SVP usage is associated with a series of mental problems, such as time distortion (Yang et al., 2024), loneliness (Wen et al., 2024), social anxiety (Yao et al., 2023), depression (Qu et al., 2024; Zhu et al., 2024), and even short video addiction (Ye et al., 2025).

Despite the growing evidence that overuse of SVPs may lead to negative impacts on mental health in the general populations, the possible neural basis of such influences remains unclear. Functional magnetic resonance imaging (fMRI) has offered a promising and non-invasive approach for characterizing between-subject differences in brain functions in the past two decades (Cao et al., 2014; Long et al., 2023). Using fMRI, many common psychiatric symptoms and disorders have been proved to be associated with significant changes in the functional brain organizations, such as depression (Tan et al., 2023; Long et al., 2020a), bipolar disorder (Long et al., 2020b), schizophrenia (Lin et al., 2021), and autism spectrum disorder (Zhang H. et al., 2024). Several substance (Huang X. et al., 2021) and non-substance (Liu et al., 2022) addictions were also associated with significantly altered brain functions as revealed by fMRI scanning. Therefore, as a specific form of digital addiction, the overuse of SVPs might also be associated with changes in certain neural circuits in the brain (Gao et al., 2025), and some studies have been performed (Gao et al., 2025; Su et al., 2021a) to test such a hypothesis. For example, Su et al. (2021a) found that viewing personalized video clips recommended by TikTok (in contrast to viewing non-personalized ones) would lead to significantly higher brain activations in the default-mode network of the brain, by performing an experiment to collect participants’ fMRI scans at the same time they watched videos in a fMRI scanner. Another study reported that viewing personalized videos recommended by SVPs will enhance the activity and connectivity of brain pathways involving the attention, frontal–parietal, and default-mode networks (Su et al., 2021b). In some other studies, the usage intensity of SVPs was associated with heightened spontaneous activity in the prefrontal cortex, posterior cingulate cortex, and temporal pole (Gao et al., 2025), as well as disrupted interactions between default-mode and control networks (Zhai et al., 2025). Collectively, these studies have provided foundational insights into how SVP usage modulates brain function and connectivity.

However, the current body of fMRI research on SVPs remains limited in several critical aspects. First, the majority of existing studies have been conducted under task-based conditions, such as having participants view personalized video clips (Su et al., 2021a; Su et al., 2021b). While informative, this approach seldom explores the brain’s activity and functional connectivity at rest. Resting-state fMRI, which is thought to reflect the brain’s intrinsic functional architecture (Tan et al., 2023; Yang et al., 2021), could serve as a valuable complement to task-based paradigms. It may help determine whether SVP usage induces long-term alterations in the brain’s spontaneous activity and functional organization, potentially persisting even when individuals are not actively engaged with video content. Therefore, investigating the effects of SVPs on brain functions using the resting-state fMRI could address this gap.

Second, we have observed that previous investigations on SVPs have primarily concentrated on examining localized brain activity or connectivity within specific regions or pathways. While this focus has yielded important insights, it is increasingly recognized that brain dysfunctions often manifest as distributed alterations across the entire brain’s network topology (Tan et al., 2023; Yang et al., 2021; Huang et al., 2025). Metrics such as the clustering coefficient, global efficiency, and local efficiency can reliably quantify the disturbances of larger-scale brain systems, potentially offering a more comprehensive perspective on brain dysfunction (Long et al., 2023; Braun et al., 2012). To our knowledge, however, it remains largely unknown whether the usage intensity of SVPs is associated with changes in these global topological properties. Therefore, future research that integrates the examination of local neural activity with the assessment of large-scale network topology would represent a valuable and necessary extension of the current literature.

Thus, to address the above gaps, this study employed resting-state fMRI to test the hypothesis that intensive SVP use is associated with altered intrinsic brain function, even in the absence of active engagement. We recruited a total of 55 healthy young adults and acquired their resting-state fMRI data. For each participant, brain functional features were quantified at multiple scales from local, network to global levels. Group comparisons on brain functional features were then conducted based on self-reported SVP usage intensity. We anticipate that our findings will provide valuable evidence to offer new insights into the neural correlates of SVP usages.

## 2 Materials and methods

### 2.1 Participants and assessments

The study initially recruited a total of sixty-two participants from Changsha, Hunan, China. All enrolled individuals met these inclusion criteria: (1) age between 18 and 25; (2) native Chinese language background; (3) absence of any severe psychiatric disorders; and (4) no contraindications to fMRI experiments. All subjects provided written informed consent, and the study received approval from the Ethics Committee of the Second Xiangya Hospital (ethics approval number 2024013). Seven participants were excluded due to poor fMRI data quality (see Section 2.2); the final analysis therefore included 55 participants (21.60 ± 1.51 years old in average; 39 females and 16 males).

Consistent with prior research (Ye et al., 2025), we assessed the intensity of each participant’s SVP usage using a five-point scale. This was based on their self-reported average daily SVP usage time, categorized into the following ranges: less than 1 h, 1–2 h, 2–3 h, 3–5 h, and more than 5 h (Ye et al., 2025). In the current study, the reported SVP usage data revealed a skewed distribution, with 20 individuals at <1 h, 24 at 1–2 h, and 11 at 2–3 h; no participants reported over 3 h of daily SVP use. Given such a distribution, to mitigate the reduced statistical power associated with multiple small subgroups and to create a clinically meaningful contrast between lower and higher users, we consolidated the cohorts into two groups: a lower SVP usage (SVP-) reference group (< 1 h, *n* = 20) and a consolidated higher SVP usage (SVP+) group (≥1 h, *n* = 35). Subsequent analyses compared demographic and neuroimaging characteristics between these two groups. It should be noted that the ≥1 h threshold does not imply pathological or addictive use; rather, it distinguishes relatively higher from lower usage within the typical range reported in the general population.

To assess participants’ recent mental health status, all subjects also completed the following self-report scales referencing the past 2 weeks: the 9-item Patient Health Questionnaire (PHQ-9) for depressive symptoms (Kroenke et al., 2001) and the 7-item Generalized Anxiety Disorder scale (GAD-7) for anxiety symptoms (Spitzer et al., 2006). The Chinese versions of these two scales have been validated and widely used in previous studies (Wu Z. et al., 2022; Zhang J. et al., 2024; Zhang et al., 2023). Both scales demonstrated good internal consistency in our sample: the Cronbach’s *α* coefficients were 0.883 for the PHQ-9 and 0.874 for the GAD-7, both exceeding the conventional threshold of 0.70 (Wu Z. et al., 2022).

### 2.2 Neuroimaging data collection and preprocessing

Resting-state fMRI and T1-weighted structural images (for registration) were collected from each participant on a 3.0 T Siemens scanner. During the scan, all participants were instructed to remain awake. Key resting-state fMRI parameters included a matrix size of 64 × 64, 32 slices, repetition time (TR) of 2000 ms, and 216 total volumes. Corresponding T1-weighted images were acquired with a 256 × 256 matrix, 176 slices, and a TR of 1900 ms. Preprocessing of all images was conducted using the DPARSF software (Yan et al., 2016; Chao-Gan and Yu-Feng, 2010), following a standard pipeline that involved removal of the first 10 time points, slice timing correction, realignment, normalization, temporal filtering, and nuisance regression (including the white matter and cerebrospinal fluid signals, as well as Friston-24 head motion parameters). We did not perform global signal regression considering that it remains a debated practice (Aquino et al., 2020; Li et al., 2026). Rigorous quality control was implemented: all preprocessed images were manually inspected for artifacts; imaging data with mean framewise displacement (FD) > 0.2 mm were excluded for excessive head motion (Long et al., 2020a); and mean FD was further included as a covariate in subsequent analyses to control for residual motion effects. The mean FD was < 0.2 mm in both groups, and the percentage of frames with FD > 0.2 mm did not differ significantly between groups (Table 1). Therefore, no scrubbing or censoring of high-motion frames was performed; instead, the Friston-24 motion parameters were included as nuisance regressors to control for potential motion effects. During these steps, a total of seven participants were excluded due to detected artifacts or excessive head motion. Further details about the scanning parameters (Huang D. et al., 2021) and the standard preprocessing pipeline (Long et al., 2020a; Long et al., 2024) are available in prior publications.

**Table 1:** Comparisons on demographic and clinical characteristics between the groups.

Characteristic	SVP+	SVP-	Group comparisons
Participants	35	20	/
Age (years)	21.51 ± 1.60	21.40 ± 1.52	*t* = 0.263, *p* = 0.794
Sex (male/female)	13/22	5/15	*χ*^2^ = 0.852, *p* = 0.356
Years of education	15.60 ± 1.38	15.90 ± 2.45	*t* = −0.541, *p* = 0.591
Mean FD (mm)	0.07 ± 0.03	0.06 ± 0.03	*t* = 0.468, *p* = 0.641
Percentage of frames with FD > 0.2 mm	0.171 ± 0.167	0.141 ± 0.134	*t* = 0.687, *p* = 0.495
GAD-7 score	3.71 ± 4.43	3.50 ± 2.26	*t* = 0.201, *p* = 0.841
PHQ-9 score	5.17 ± 5.19	4.30 ± 3.48	*t* = 0.669, *p* = 0.507

SVP+, the higher short-video-platforms usage group; SVP-, the lower short-video-platforms usage group.

### 2.3 Brain functional feature extractions

To comprehensively characterize the neural correlates of SVP usage, we estimated and compared brain functional features between the lower and higher SVP usage groups across three distinct spatial scales: voxel-level, network-level, and whole-brain level. At the most granular, voxel-level, we computed two measures reflecting local brain activity: (1) the Amplitude of Low-Frequency Fluctuations (ALFF) and fractional ALFF (fALFF), which quantify the intensity of spontaneous neural activity within a single voxel (Li et al., 2024; Fortier et al., 2024); and (2) Regional Homogeneity (ReHo), which measures the temporal synchronization of a given voxel with its nearest neighbors, serving as an index of local functional coherence (Zhou et al., 2024; Han et al., 2025).

**Figure 1:**
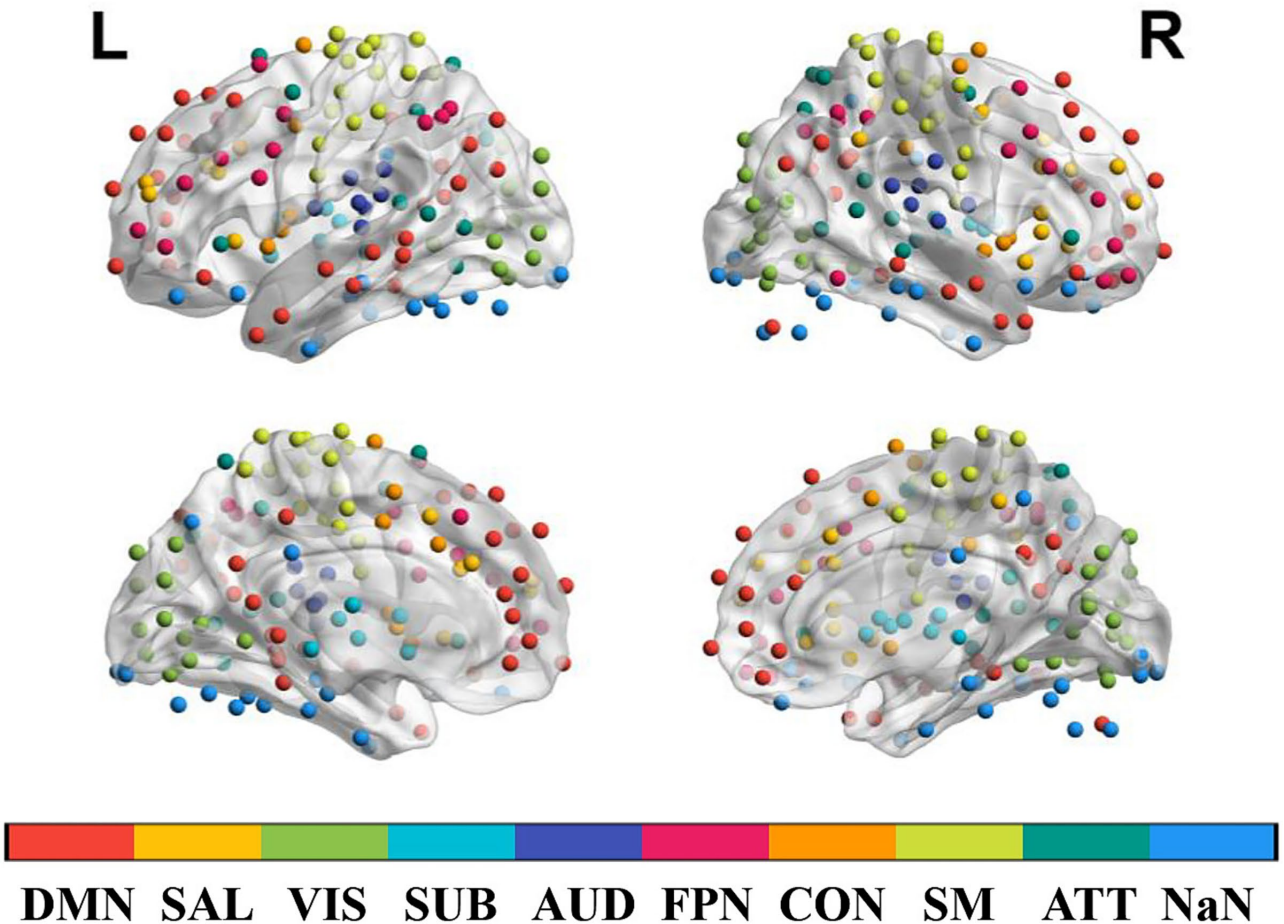
The 264 regions of interest defined in the present study and their brain network assignments. ATT, attention network; AUD, auditory network; CON, cinguloopercular network; DMN, default-mode network; FPN, frontoparietal network; L, left hemisphere; NaN, unassigned; R, right hemisphere; SAL, salience network; SM, sensorimotor network; SUB, subcortical network; VIS, visual network.

Subsequently, the brain was parcellated into distinct functional networks using the Power atlas (Power et al., 2014), which provided a framework for our subsequent network-level and global-level analyses. Specifically, a total of 264 regions of interest (ROIs) were defined and assigned to nine major brain networks, including the default-mode, frontoparietal, salience, cingulo-opercular, visual, auditory, sensorimotor, attention, and subcortical networks (Figure 1) (Long et al., 2023; Li et al., 2023; Ouyang et al., 2022). At the network level, we further investigated the functional integrations between these large-scale brain networks. This was achieved by first calculating the edge-level functional connectivity (FC) between all ROIs, defined as the temporal correlation of the BOLD signal time series averaged within each ROI. Negative FC (correlation coefficients) were retained as originally computed (i.e., not converted to absolute values or set to zero). Based on the resulting FC matrix, we then examined both intra-network connectivity (by averaging FC between ROIs within each predefined functional network) and inter-network connectivity (by averaging FC between ROIs belong to a pair of distinct networks) (Long et al., 2024).

Finally, at the global level, we employed graph theory to model the brain as a complex network. From this model, we extracted a set of key topological metrics to quantify the brain’s global integration and segregation efficiency. These included: global efficiency, reflecting the overall capacity for parallel information transfer across the entire network; local efficiency, indicating the fault tolerance and information processing capability within localized neighborhoods; characteristic path length, measuring the average efficiency of traversing the network; and the clustering coefficient, quantifying the extent of local interconnectedness or cliquishness (Zhou and Long, 2024; Rubinov and Sporns, 2010). All these topological network metrics were computed from the weighted FC matrices based on the 264 Power’s ROIs, using the DPABINet toolbox (Yan et al., 2024). To avoid relying on a single sparsity threshold, we constructed graphs over a continuous range of network densities from 0.1 to 0.34 (in steps of 0.01). This density range allows the thresholded networks to be estimable for graph metrics while limiting spurious edges (Achard and Bullmore, 2007; van Wijk et al., 2010). For each graph metric, we computed the area under the curve (AUC) across all densities, which was then used for statistical comparisons between groups. It is noteworthy that negative correlation coefficients were not explicitly excluded; however, typically only the strongest positive edges were retained during thresholding (0.1–0.34), and as a result negative edges rarely entered the graph analysis. Therefore, the treatment of negative edges is unlikely to substantially affect our results.

### 2.4 Statistics

Mean age, sex ratio, years of education, head motion (mean FD), as well as scores of the PHQ-9 and GAD-7 scales were compared between the SVP- and SVP + groups using t-tests or Chi-square tests as appropriate. To identify regional brain activity differences, the ALFF, fALFF, and ReHo values were compared between the two groups using two-sample t-tests that included age, sex, years of education, and mean FD as covariates of no interest; statistical significance was set at AlphaSim-corrected *p* < 0.001. Edge-level FC was compared between groups using the Network-Based Statistic (NBS) approach (Zalesky et al., 2010) (5,000 iterations) with age, sex, years of education, and head motion as covariates; significance was set at edge *p* < 0.001 and cluster *p* < 0.05 after NBS corrections. Network-level FC and global topological metrics were compared between groups using the Analysis of Covariance (ANCOVA) covarying for age, sex, years of education, and head motion; the results were considered significant at *p* < 0.05 after false discovery rate (FDR) corrections across multiple tests (e.g., across the 45 network-level FC values).

### 2.5 Supplementary analyses

Following the main analyses, several additional supplementary analyses were performed. First, to test whether our results were robust to different parcellations, we repeated the analyses on functional connectivity (FC) and graph metrics using an independent functional atlas (the Dosenbach atlas) (Dosenbach et al., 2010). Second, to further test the potential influence of residual motion, we repeated all main analyses (voxel-wise measures, network-level FC, edge-level FC, and global graph metrics) by adding the percentage of frames with FD > 0.2 mm as an additional covariate alongside mean FD. Third, although not examined in our primary analyses, we also computed normalized small-world indices (sigma, gamma, lambda) as supplementary analyses to further characterize network topology. For each density from 0.1 to 0.34 (step = 0.01), gamma and lambda were derived by comparing real networks with 100 random networks; sigma was calculated as gamma/lambda. Group differences were assessed using the same statistical methods as in the primary analyses. Fourth, for any brain functional measures showing significant between-group differences, post-hoc partial Spearman’s correlations with age, sex, years of education, and mean FD as covariates were performed to examine their relationships with PHQ-9/GAD-7 scores.

## 3 Results

### 3.1 Demographic and clinical characteristics

Comparisons of demographic/clinical characteristics between the SVP + and SVP- groups are presented in Table 1. There were no significant differences between the two groups in age, sex, years of education and head motion (all *p* > 0.05). In addition, there were no significant differences in the GAD-7 and PHQ-9 total scores between the groups (both *p* > 0.05).

### 3.2 Voxel-level measurements

**Figure 2:**
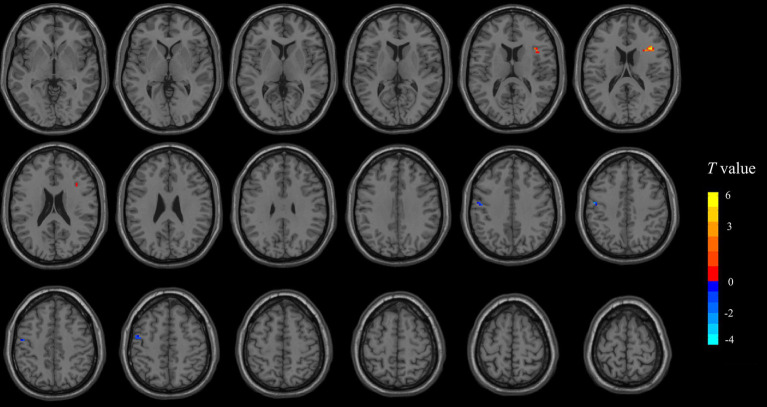
The two clusters with significant between-group differences in the fALFF values. The clusters showing elevated and reduced fALFF in the SVP + group are presented by the blue and red-yellow colors, respectively. More detailed information about the two clusters can be found in Table 2.

At the voxel level, significant differences in the fALFF were observed between the two groups (*p* < 0.001, AlphaSim-corrected) (Figure 2 and Table 2). The analysis identified two separate clusters where these differences were localized: one in the left precentral gyrus, where the SVP + group demonstrated higher fALFF than the SVP- group; and another in the right frontal lobe, specifically the opercular part of the inferior frontal gyrus, where the SVP + group showed lower fALFF. In contrast to the fALFF findings, neither the ALFF nor ReHo maps yielded any statistically significant difference between groups in any brain region.

**Table 2:** Information on the two clusters with significant between-group differences in the fractional amplitude of low-frequency fluctuations (fALFF).

Cluster	Group comparison	AAL3	Brodmann Atlas	Number of voxels	MNI coordinate of peak	*t* value of peak	*p* value of peak
Cluster 1	SVP- > SVP+	Frontal_Inf_Oper_R	BA48_R	20	36, 18, 18	5.045	< 0.001
Cluster 2	SVP- < SVP+	Left Precentral_L	BA6_L	22	−51, −9, 42	−4.823	< 0.001

AAL, automated anatomical atlas; MNI, Montreal Neurological Institute; SVP+, the higher short-video-platforms usage group; SVP-, the lower short-video-platforms usage group.

### 3.3 Edge- and network-level connectivity

**Figure 3:**
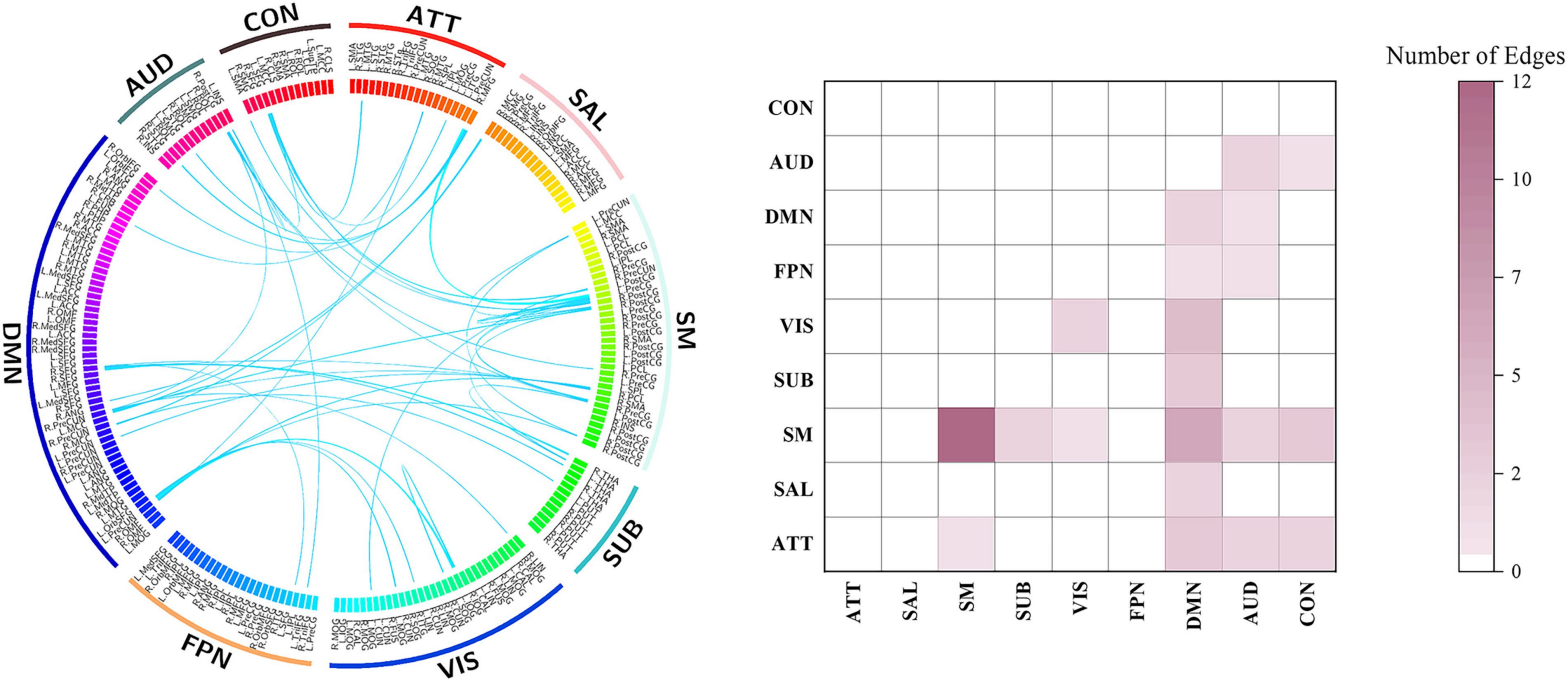
The edges with significant increased FC (corrected *p* < 0.05) in the SVP + than SVP- group. ATT, attention network; AUD, auditory network; CON, cingulo-opercular network; DMN, default-mode network; FPN, frontoparietal network; SAL, salience network; SM, sensorimotor network; SUB, subcortical network; VIS, visual network.

Compared to the SVP- group, the SVP + group exhibited significant increases in both the edge- and network-level FCs (corrected *p* < 0.05). The edges with significantly higher FCs in the SVP + than SVP- group were displayed in Figure 3; for clearer visualization, we counted and presented the number of edges belonging to each of the intra- and inter-network categories. These edges involved most major brain networks, including the sensorimotor, visual, auditory, subcortical, default-mode, attention, and cingulo-opercular networks (Figure 3).

**Figure 4:**
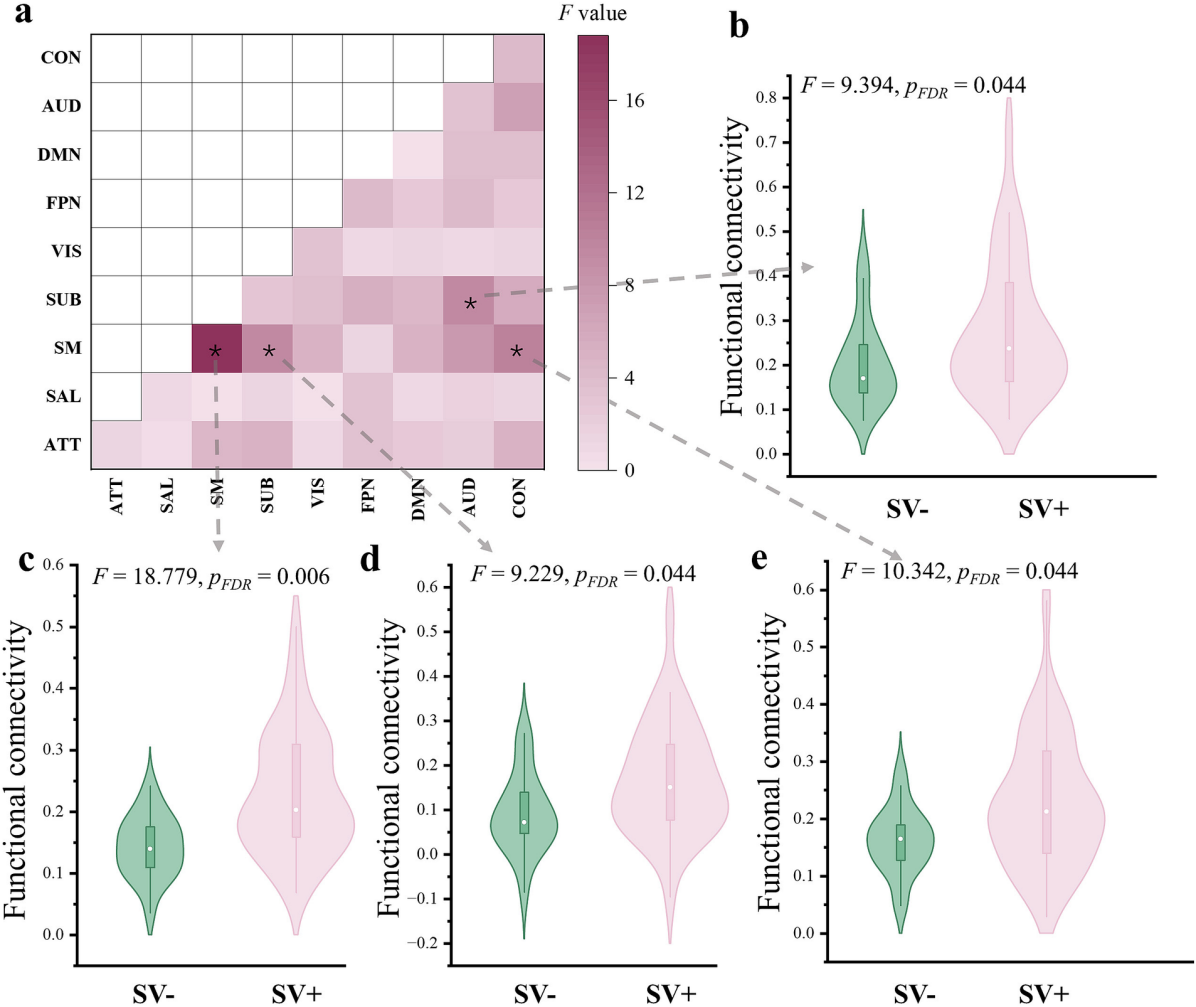
Results of comparisons on network-level FC between groups. The “*” indicates a significant difference with corrected *p* < 0.05. ATT, attention network; AUD, auditory network; CON, cingulo-opercular network; DMN, default-mode network; FPN, frontoparietal network; SAL, salience network; SM, sensorimotor network; SUB, subcortical network; SVP-, the lower short-video-platform usage group; SVP+, the higher short-video-platform usage group; VIS, visual network.

The results of between-group comparisons on network-level FCs were presented in Figure 4. Significantly higher FC (corrected *p* < 0.05) was observed in the SVP + group within the sensorimotor network, between the sensorimotor and subcortical networks, between the sensorimotor and cinguloopercular networks, as well as between the auditory and subcortical networks. Thus, after averaging across each network, group differences in these networks remained particularly prominent.

### 3.4 Global topological metrics

**Figure 5:**
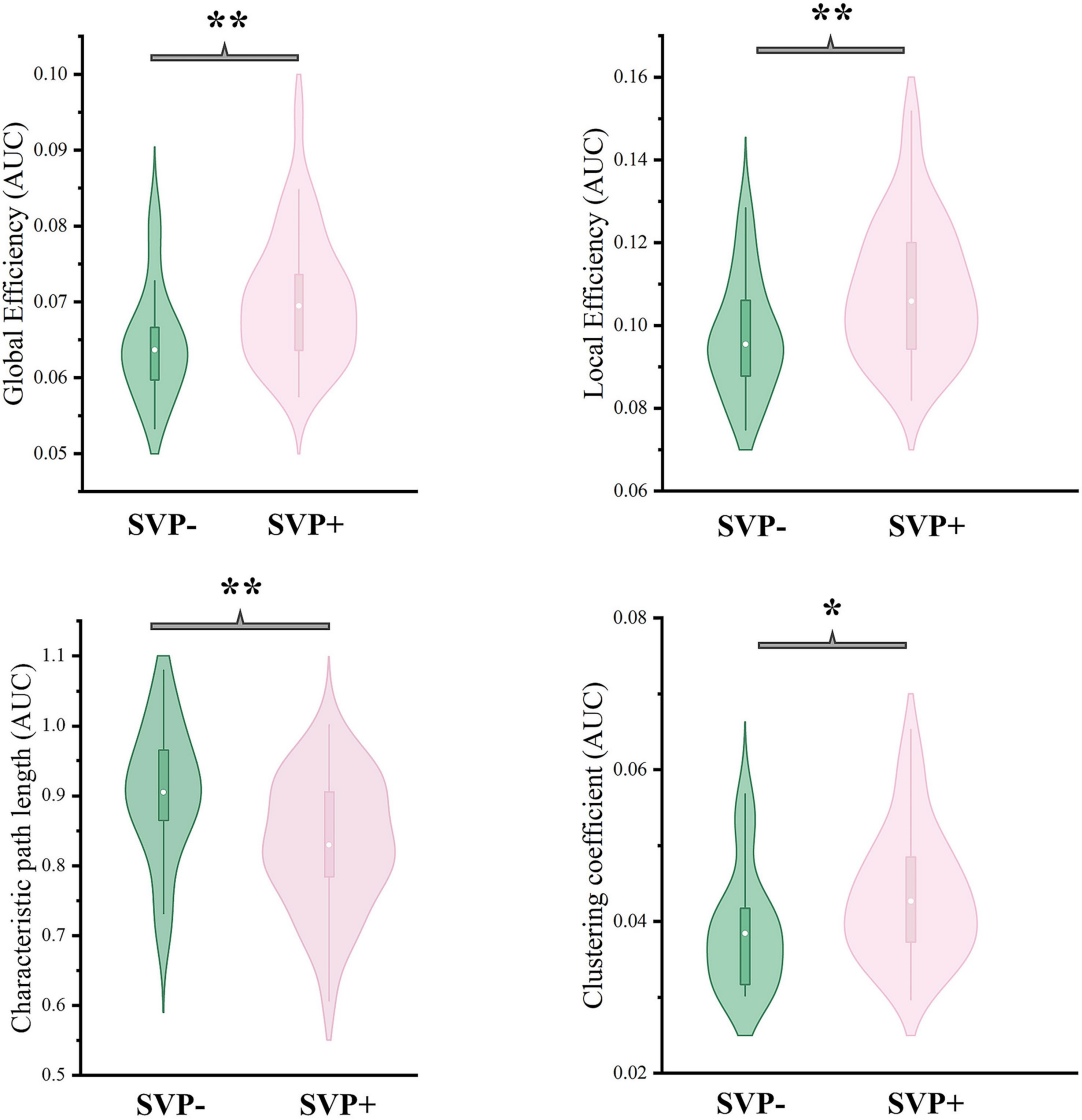
Results of comparisons on global topological metrics between groups. *corrected *p* < 0.05; **corrected *p* < 0.01; AUC, area under curve; SVP-, the lower short-video-platform usage group; SVP+, the higher short-video-platform usage group.

The results of between-group comparisons on global topological metrics were presented in Figure 5. Compared to the SVP- group, the SVP + group exhibited significantly higher global efficiency (*F* = 9.188, corrected *p* = 0.007), higher local efficiency (*F* = 8.433, corrected *p* = 0.007), lower characteristic path length (*F* = 9.897, corrected *p* = 0.007), and higher clustering coefficient (*F* = 7.186, corrected *p* = 0.010).

### 3.5 Supplementary analyses

When using the Dosenbach 160 atlas, the results of FC did not survive multiple comparison corrections. However, the group differences in key graph-theory metrics (e.g., global efficiency and clustering coefficient) were highly consistent with those from our primary parcellation (corrected *p* < 0.05, see Supplementary Figure S1; Supplementary Table S1). These findings suggest that our graph-theory results are robust to the choice of parcellation.

After adding the percentage of frames > 0.2 mm as an additional covariate, edge-level FC group differences no longer survived multiple comparison correction. However, voxel-wise measures, network-level FC, and global graph metrics remained essentially unchanged (corrected *p* < 0.05, see Supplementary Figures S2, S3; Supplementary Tables S2, S3). Thus, while edge-level FC may be more sensitive to subtle motion, our core conclusions at the voxel, network, and global levels are not meaningfully confounded by high-motion frames.

No significant group differences were observed for the normalized small-world indices sigma, gamma, or lambda (all *p* > 0.05).

In post-hoc correlation analyses, except for the clustering coefficient, which showed a modest positive correlation with PHQ-9 scores (rho = 0.306, *p* = 0.029; see Supplementary Figure S4), no other significant correlations were observed between any measure and either clinical scale (all *p* > 0.05).

## 4 Discussion

In this study, we investigated the possible association between usage intensity of SVPs and altered brain function using the resting-state fMRI method. The results revealed significant differences between the SVP- and SVP + groups in brain functional measures across multiple levels of analysis, which can offer valuable insights into the neural correlates of SVP usages.

At the level of single edges, the SVP + group exhibited significantly heightened FCs across numerous edges linking most major brain networks (Figure 3). These included the sensorimotor network, which is responsible for processing and integrating sensory and motor information (Zhao et al., 2025); the visual and auditory networks, which is thought to be crucial for perceptual processing (Noyce et al., 2022); the subcortical network, which is key for relaying and modulating information passing to different cortical networks (Long et al., 2023); the default-mode network, known to medicate one’s self-referential thought and internally directed processing (Tang et al., 2022); as well as the attention and cingulo-opercular networks, which is related to higher-order cognitive controls (Robinson et al., 2025). Analyses on the network-level FCs, which aggregate and average FC strengths within each predefined network, corroborated this trend of widespread hyperconnectivity (Figure 4). Notably, after applying rigorous statistical corrections for multiple comparisons, the enhanced FCs within the sensorimotor, auditory, subcortical, and cingulo-opercular networks remained particularly robust (corrected *p* < 0.05). Complementing the above connectivity findings, voxel-wise analysis of the fALFF identified a specific cluster in the left precentral gyrus (a core component of the sensorimotor cortex) (Chen et al., 2024) with significantly higher fALFF in the SVP + group, which suggests an elevated regional spontaneous neural activity (Figure 2). Collectively, these results paint a picture of a brain in the SVP + group characterized by a generally elevated state of functional connections and local spontaneous activity, with the sensorimotor system appearing as a prominent locus of change. It is important to note, however, that this pattern was not universally increased, as a separate cluster showing reduced fALFF was addionally detected in the right frontal lobe, specifically the opercular part of the inferior frontal gyrus (Figure 2), hinting at a more complex, cortex-specific pattern of alteration rather than a monolithic shift.

Our observations in the resting state resonate with and extend prior research mostly conducted under task-based conditions. For example, a previous study demonstrated that viewing personalized video content recommended by SVPs will acutely enhances activity and connectivity within pathways linking the attention and default-mode networks (Su et al., 2021b). Other studies have correlated SVP usage with increased spontaneous activity in regions such as the prefrontal cortex, posterior cingulate cortex, and temporal pole (Gao et al., 2025). Our study consolidates these findings and demonstrates that similar patterns of hyperconnectivity and altered activity can be detectable in the absence of an explicit task. Moreover, we also found some patterns associated with SVP usage that were not previously reported in task-based research, such as increased FC in sensorimotor and subcortical networks, suggesting that resting-state fMRI may reveal unique neural alterations beyond those captured during active task performance. Taking these together, we can hypothesize that SVP usage may not only transiently modulate brain function during use but may also be associated with longer-term adaptations in the brain’s intrinsic functional organization. These alterations may persist as a trait-like feature, potentially influencing cognitive and affective processes even when the individual is not actively engaged with video content. However, it should be noted that we did not assess the total duration of relatively higher-intensity SVP habits; thus, the hypothesis of longer-term adaptations requires direct validation in future studies.

Beyond regional and network-specific changes, we explored the impact of SVP usage on the global topological properties of the whole-brain functional network. Graph theory analyses revealed that the SVP + group exhibited a configuration characterized by significantly higher global efficiency, higher local efficiency, higher clustering coefficient, and lower characteristic path length (Figure 5). Such a shift is indicative of an altered topology with higher efficiency and clustering than that of the SVP- group (Zhou and Long, 2024). While the small-world architecture is considered important for balancing information segregation and integration, a shift toward an excessively increased or decreased efficiency is thought to be abnormal and was reported in multiple psychiatric disorders (Suo et al., 2018). To our knowledge, prior neuroimaging literature has seldom investigated such global network metrics in the context of SVP usage. Therefore, our study addresses a notable gap, providing initial evidence that relatively higher-intensity SVP engagement might be associated with a reconfiguration of the brain’s functional topology at a global scale.

The patterns of functional alteration observed in our SVP + group, such as altered network-level FCs and shifted global network topology, have been reported in several psychiatric disorders. For instance, altered FCs within and involving the sensorimotor network have been widely reported in common psychiatric disorders such as schizophrenia (Wu G. et al., 2022), major depressive disorder (Long et al., 2024), and bipolar disorder (Zhu et al., 2022). Furthermore, a shifted small-world topological organization has been previously documented in patients with schizophrenia (Yu et al., 2013) and post-traumatic stress disorder (Lei et al., 2015). This convergence might not directly imply that higher-intensive SVP users have a psychiatric condition; indeed, our clinical scores of depressive and anxiety symptoms did not reach significant group differences (Table 1), and no significant correlations were found between most brain functional measures and clinical symptoms. However, it raises the possibility that the neural correlates associated with higher SVP usage may partly overlap with the substrates of certain mental health vulnerabilities. These shared functional signatures could represent a potential neural mechanism partly explaining the epidemiological associations frequently reported between excessive social media/digital media use and poorer mental well-being (Qu et al., 2024; Zhu et al., 2024).

On the other hand, an alternative interpretation should be considered. In the neuroimaging literature, altered network topology with higher efficiency has also been associated with superior cognitive performance and efficient information transfer. For example, studies have linked higher global efficiency to higher general intelligence and better working memory (van den Heuvel et al., 2009; Langer et al., 2012; Barbey, 2018; Stanley et al., 2015). Thus, the observed topological alterations in the SVP + group might instead reflect a form of neural adaptation or enhanced information-processing capacity, possibly resulting from intensive engagement with fast-paced, attention-demanding video content. Unfortunately, we did not collect cognitive or behavioral measures (e.g., processing speed or attention tasks) in this cohort, which prevents us from directly testing this hypothesis. Future studies should incorporate such measures to determine whether the altered brain network topology associated with a relatively higher SVP usage confers cognitive benefits or represents a risk factor for maladaptive outcomes.

Our study has some limitations. First, our sample size is relatively small, and the study design is cross-sectional. This may limit the statistical power to detect more subtle effects (e.g., possible differences between the 1–2 h and 2–3 h groups), and limit our ability to establish causal relationships between SVP usage and brain functional changes. Second, our study solely collected resting-state fMRI scans, without any SVP-related task conditions for comparisons. Third, while our analyses were performed under a conventional framework of “static” functional brain network, recent studies have suggested that brain organizations can fluctuate over time, and analyses of “dynamic FC” may provide information ignored by conventional methods (Long et al., 2023). Thus, future studies using dynamic FC analyses may be needed. Another limitation is that we used AlphaSim correction for voxel-wise measures rather than a more conservative nonparametric method (e.g., TFCE), which typically requires larger sample sizes. Due to our modest sample size, we did not apply nonparametric correction; future studies with larger cohorts should validate our findings using such approaches. Finally, it should be noted that we did not collect information on several acute state variables that may affect brain functions, including time-of-day of scanning, recency of SVP use, and caffeine or nicotine intake. We also did not collect the total duration (months/years) of high-intensity SVP habit, which may help distinguish between recent behavior and long-term neuroplasticity. These factors could potentially confound the observed group differences, and future studies are warranted to systematically control for them.

## 5 Conclusion

In conclusion, this study provides multi-level resting-state fMRI evidence suggesting that a relatively higher-intensity SVP usage is associated with a distinct pattern of brain functional organization, characterized by widespread hyperconnectivity involving most major brain networks, localized spontaneous activity alterations in sensorimotor regions, and a shift toward an excessively strong small-world network topology. These findings align with and extend prior task-based studies, possibly supporting the hypothesis that SVP usage is associated with long-term alterations in the brain’s intrinsic functional architecture. Our study highlights the importance of considering possible neurocognitive impacts of SVP usage, and calls for future larger-sample and longitudinal studies to further understand such relationships.

## Data Availability

The raw data supporting the conclusions of this article will be made available by the authors, without undue reservation.
