# Supplementary material for: Association between usage intensity of short video platforms and altered brain function: a resting-state functional magnetic resonance imaging study
Source: Front Hum Neurosci. 2026 May 21;20:1786568. doi: 10.3389/fnhum.2026.1786568 (PMC13233705; doi:10.3389/fnhum.2026.1786568)

# Association between usage intensity of short video platforms and altered brain function: a resting-state functional magnetic resonance imaging study

***Supplemental Information (SI)***

## SI Results

### Supplementary analyses

When using the Dosenbach 160 atlas, the results of functional connectivity (FC) did not survive multiple comparison corrections. However, the group differences in key graph‑theory metrics (e.g., global efficiency and clustering coefficient) were highly consistent with those from our primary parcellation (**Supplementary Figure 1** and **Supplementary Table 1**).

After adding the percentage of frames with FD (framewise displacement) > 0.2 mm as an additional covariate, edge‑level FC group differences no longer survived multiple comparison correction. However, voxel‑wise measures, network‑level FC, and global graph metrics remained essentially unchanged (see **Supplementary Figures 2-3** and **Supplementary Tables 2-3**).

In post-hoc correlation analyses, except for the clustering coefficient, which showed a modest positive correlation with PHQ-9 scores (rho = 0.306, *p* = 0.029; see **Supplementary Figure S4**), no other significant correlations were observed between any measure and either clinical scale (all *p* > 0.05).

**Supplemental Table S1.** Group comparisons of graph metrics with the Dosenbach 160 Atlas. SVP-, the lower short-video-platform usage group; SVP+, the higher short-video-platform usage group.

| **Metrics** | **SVP-** | **SVP+** | ***F* value** | ***p*** |
| --- | --- | --- | --- | --- |
| Global Efficiency | 0.064 ± 0.007 | 0.070 ± 0.008 | 6.66 | 0.013* |
| Local Efficiency | 0.097 ± 0.014 | 0.109 ± 0.017 | 6.40 | 0.015* |
| Clustering Coefficient | 0.039 ± 0.008 | 0.043 ± 0.008 | 4.97 | 0.030* |
| Characteristic path length | 0.905 ± 0.094 | 0.832 ± 0.092 | 6.97 | 0.011* |

*** corrected *p* < 0.05.**

**Supplemental Table S2.** Group comparisons of graph metrics after adding the percentage of frames with FD > 0.2 mm as an additional covariate. SVP-, the lower short-video-platform usage group; SVP+, the higher short-video-platform usage group.

| **Metrics** | **SVP-** | **SVP+** | ***F* value** | ***p*** |
| --- | --- | --- | --- | --- |
| Global Efficiency | 0.064 ± 0.007 | 0.070 ± 0.008 | 9.00 | 0.004* |
| Local Efficiency | 0.097 ± 0.014 | 0.109 ± 0.017 | 8.41 | 0.006* |
| Clustering Coefficient | 0.039 ± 0.008 | 0.043 ± 0.008 | 5.90 | 0.019* |
| Characteristic path length | 0.905 ± 0.094 | 0.832 ± 0.092 | 9.61 | 0.003* |

*** corrected *p* < 0.05.**

**Supplemental Table S3.** Information on the three clusters with significant between-group differences in the fractional amplitude of low-frequency fluctuations (fALFF) after adding the percentage of frames with FD > 0.2 mm as an additional covariate. AAL, Automated anatomical atlas; MNI, Montreal Neurological Institute; SVP+, the higher short-video-platforms usage group; SVP-, the lower short-video-platforms usage group.

| **Cluster** | **Group comparison** | **AAL3** | **Brodmann Atlas** | **Number of voxels** | **MNI coordinate of peak** | ***t* value of peak** | ***p* value of peak** |
| --- | --- | --- | --- | --- | --- | --- | --- |
| Cluster 1 | SVP- > SVP+ | Frontal_Inf_Oper_R | BA48_R | 15 | 36, 18, 18 | 4.879 | < 0.001 |
| Cluster 2 | SVP- < SVP+ | Left Precentral_L | BA6_L | 16 | -51, -9, 42 | -4.591 | < 0.001 |
| Cluster 3 | SVP- > SVP+ | Cingulate_Mid_R | NA | 17 | 25, 36, 37 | 4.242 | < 0.001 |

**Supplemental Figure S1** Group comparisons of graph metrics with the Dosenbach 160 Atlas. *corrected *p* < 0.05; AUC, area under curve; SVP-, the lower short-video-platform usage group; SVP+, the higher short-video-platform usage group.


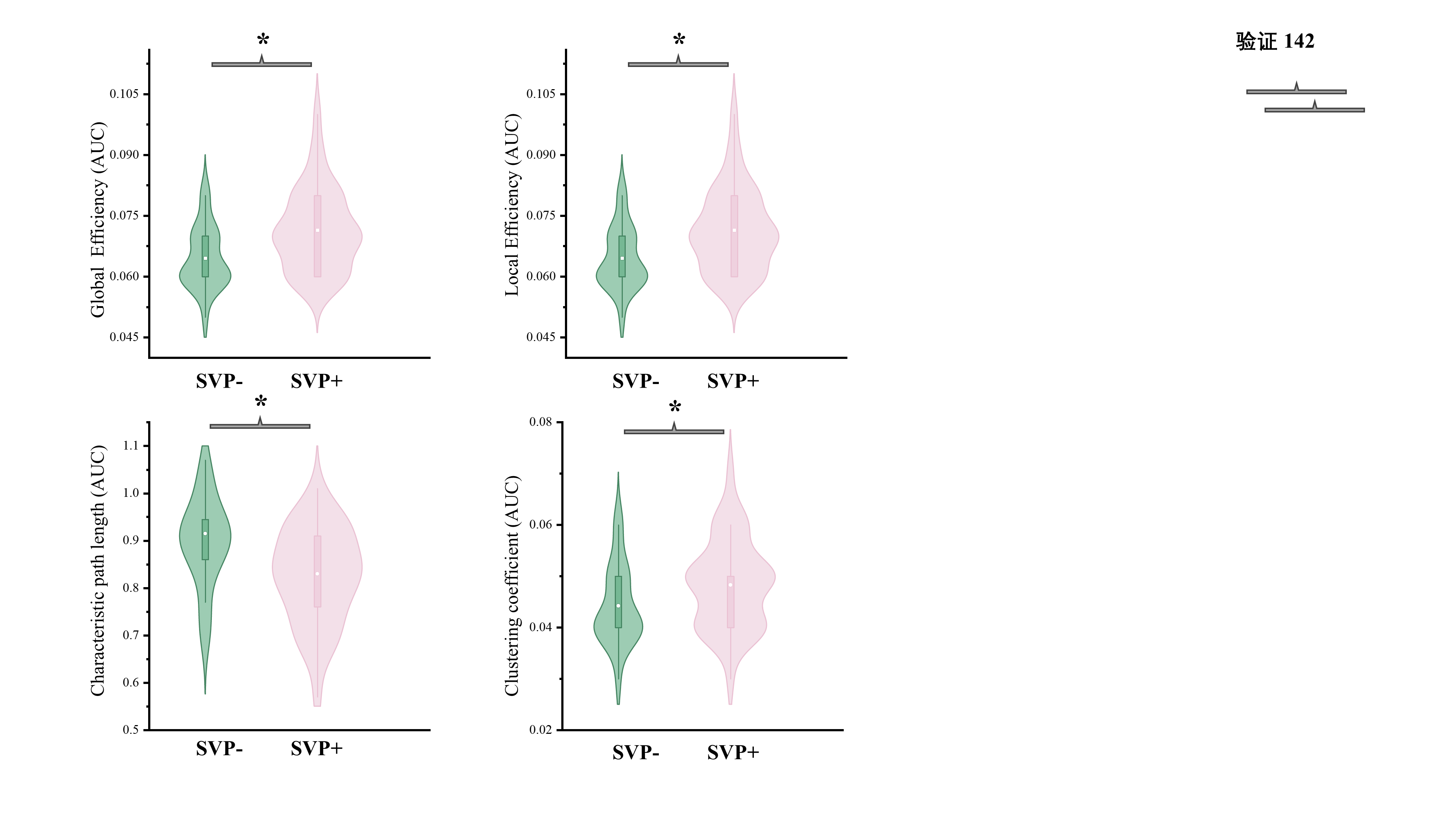


**Supplemental Figure S2** Group comparisons of network-level FC after adding the percentage of frames with FD > 0.2 mm as an additional covariate. The “*” indicates a significant difference with corrected *p* < 0.05. ATT, attention network; AUD, auditory network; CON, cingulo-opercular network; DMN, default-mode network; FPN, frontoparietal network; SAL, salience network; SM, sensorimotor network; SUB, subcortical network; SVP-, the lower short-video-platform usage group; SVP+, the higher short-video-platform usage group; VIS, visual network.

**
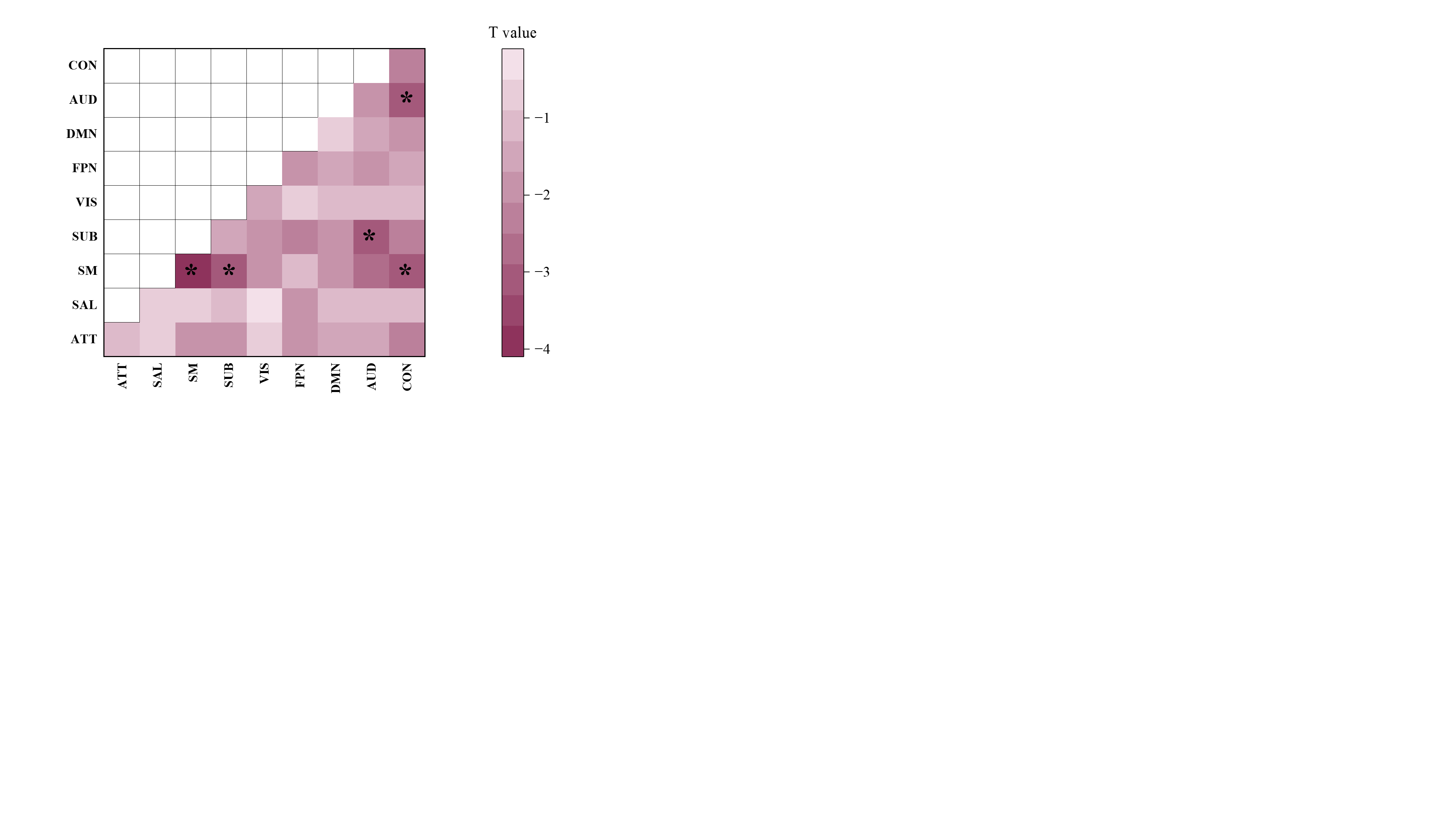
**

**Supplemental Figure S3** The clusters with significant between-group differences in the fALFF values after adding the percentage of frames with FD > 0.2 mm as an additional covariate. The clusters showing elevated and reduced fALFF in the SVP+ group are presented by the blue and red-yellow colors, respectively. More detailed information about the two clusters can be found in **Supplemental Table S3**.


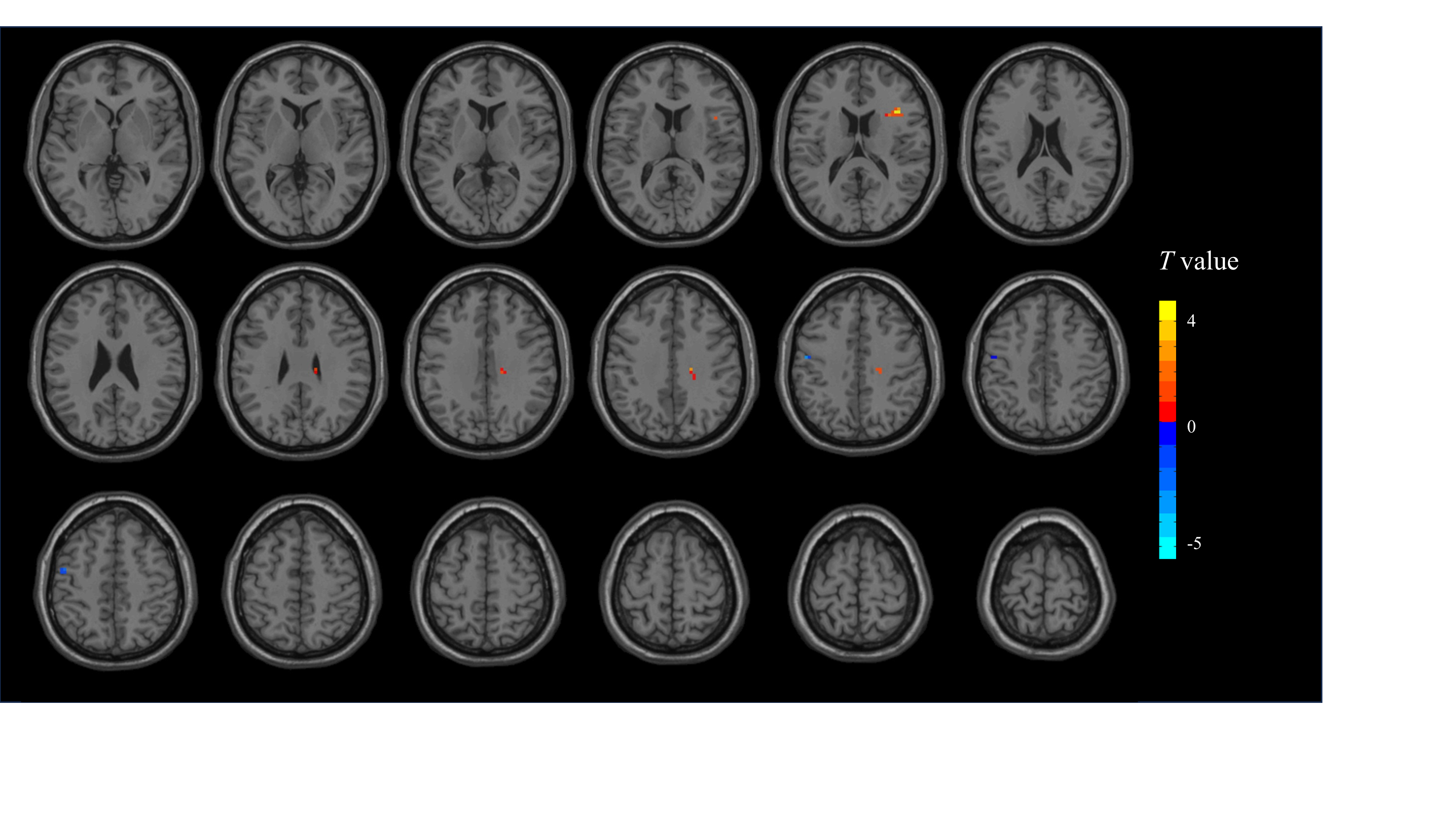


**Supplemental Figure S4** Partial spearman’s correlation between the clustering coefficient and PHQ-9 scores with the age, sex, years of education, and mean FD as covariates. AUC, area under curve; PHQ-9, the 9-item Patient Health Questionnaire.


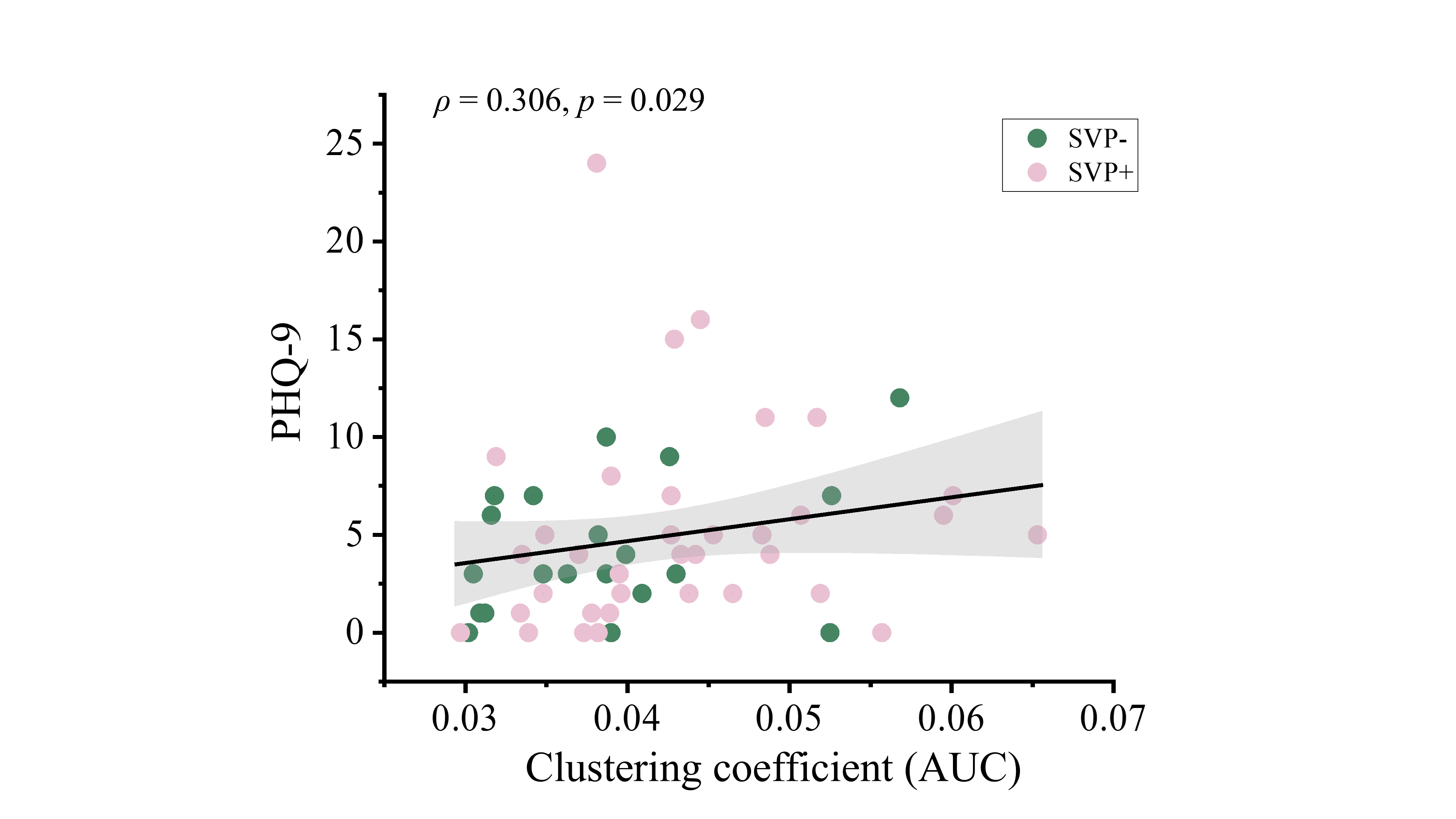

Supplement: Supplementary file 1 [file Data_Sheet_1.docx]
